# The association between physical fitness and mental health in Norwegian adolescents

**DOI:** 10.1186/s12889-020-08936-7

**Published:** 2020-05-24

**Authors:** Andreas Åvitsland, Eva Leibinger, Tommy Haugen, Øystein Lerum, Runar B. Solberg, Elin Kolle, Sindre M. Dyrstad

**Affiliations:** 1 Department of Education and Sport Science, University of Stavanger, 4036 Stavanger, Norway; 2 Department of Sport Science and Physical Education, University of Agder, 4604 Kristiansand, Norway; 3 Department of Sport, Food and Natural Sciences, Western Norway University of Applied Sciences, 6851 Sogndal, Norway; 4 Department of Sports Medicine, Norwegian School of Sports Sciences, 0806 Oslo, Norway; 5 Department of Public Health, University of Stavanger, 4036 Stavanger, Norway

**Keywords:** Physical fitness, Mental health, Strengths and difficulties, Norway, Adolescence, Cross-sectional

## Abstract

**Background:**

Studies indicate that health-related components of physical fitness are associated with mental health outcomes. However, research is scarce concerning this relationship in young adolescents in general and non-existent in Norwegian populations specifically. The aim of the study was to examine whether body composition, muscular strength and cardiorespiratory fitness were associated with self-reported mental health in Norwegian adolescents.

**Methods:**

Adolescents from four regions of Norway (*n* = 1486; mean age = 13.9; girls = 50.6%) participated. Self-reported mental health (psychological difficulties) was measured by completing the Strengths and Difficulties Questionnaire. Cardiorespiratory fitness was assessed with an intermittent running test; muscular strength was assessed by measuring handgrip strength, standing broad jump and sit-ups; and body composition was assessed by calculating body mass index from weight and height. Linear mixed effects models were conducted to assess the associations between the health-related components of physical fitness and psychological difficulties. School clusters were included as random effects and all models were controlled for sex, socioeconomic status and birthplace (domestic or foreign).

**Results:**

Body composition was not associated with psychological difficulties. Muscular strength was independently associated with psychological difficulties, but when all independent variables were entered in the fully adjusted model, only cardiorespiratory fitness was associated with psychological difficulties.

**Conclusions:**

There was a small but significant inverse association between cardiorespiratory fitness and levels of psychological difficulties in Norwegian adolescents. The results suggest that muscular strength is not associated with psychological difficulties in adolescents, when controlling for cardiorespiratory fitness. Future research should focus on the prospective association between physical fitness components and mental health outcomes in adolescents.

**Trial registration:**

The study is registered in ClinicalTrials.gov ID nr: NCT03817047. Retrospectively registered January 25, 2019.

## Background

*“Mental health is defined as a state of well-being in which every individual realizes his or her own potential, can cope with the normal stresses of life, can work productively and fruitfully, and is able to make a contribution to her or his community”* [1]. Mental health problems affect between 10 and 20% of the global child and adolescent population [2] and approximately 18% of adults will have experienced a form of mental disorder within a one-year period [3]. Depression is the most prevalent mental disorder, accounting for 41% of all disability-adjusted life years caused by mental and substance use disorders [4]. Adolescent mental health problems have increased during recent decades in middle- and high-income countries [5]. Although part of the increase may be attributed to more awareness, help-seeking, and a lower threshold for treatment, a real prevalence increase for mental health problems has likely occurred. In Norway, the percentage of lower secondary school girls reporting depressive symptoms has increased from 16% in 2011 to 20% in 2016 [6]. Furthermore, at the end of upper secondary school, 12% of boys and 29% of girls display high levels of depressive symptoms. Onset of depression during adolescence is associated with poor general health, increased work impairment and higher utilization of health care services at age 20 [7], which has contributed to mental disorders being the costliest conditions in Norway [8]. Considering these detrimental effects of adolescent mental health problems, it is important to find effective methods of prevention, or methods to improve adolescent mental health.

### Physical activity, physical fitness and potential mechanisms

Evidence suggests physical activity is a protective factor against mental health problems such as depression [9]. Moreover, physical activity can positively affect a range of other mental health outcomes, such as mood, stress, cognitive functioning [10], and self-worth [11]. Although there is no clear consensus, many mechanisms have been hypothesized to explain the relationship between physical activity and mental health. Lubans et al. [12] elucidated three of these mechanisms. First, the neurobiological mechanism proposes that physical activity alters structural and functional compositions of the brain. Second, the psychosocial mechanism proposes that physical activity can provide social interaction, physical mastery, independence and improved appearance self-perception. Lastly, the behavioral mechanism proposes that changes in behavior, such as sleep and coping skills, mediates how physical activity affects mental health outcomes.

While our physical activity level fluctuates from week to week, physical fitness, although somewhat influenced by genes [13], represents the type, frequency, intensity and duration of physical activity that has occurred over time [14]. Physical fitness may therefore provide a more stable measure of habitual physical activity levels. Physical fitness can be divided into health-related components, such as cardiorespiratory fitness (CRF), muscular strength, and body composition [15]. Given the relationship between physical activity and physical fitness, the mechanisms proposed to explain the relationship between physical activity and mental health might also apply for physical fitness and mental health [16]. However, the aforementioned components of physical fitness may also be associated with mechanisms influencing mental health outcomes, independent from physical activity [17]. High CRF, as a result of vigorous aerobic physical activity [18] can affect neurobiological processes and inhibit inflammation [16]. Body composition affects body image [19], which in turn depends on cultural norms [20]. Therefore, body composition may affect mental health outcomes through sociocultural or psychosocial mechanisms, in addition to the possible biological mechanisms that are associated with obesity [21]. Muscular strength may also depend on cultural norms [22], thereby possibly affecting mental health through similar mechanisms as body composition. Additionally, muscular strength may affect neurobiological processes differently than CRF, however, this is unclear [23].

### Mental health and physical fitness in adolescents

Many studies have been conducted with adult populations regarding associations between mental health outcomes and physical fitness. For instance, reviews show that lower levels of mental disorders have been associated with higher CRF [24], muscular strength [25] and healthier body composition [26]. Similar studies examining adolescent populations, however, are scarce. Ruggero et al. [27], showed that CRF was inversely associated with depression in 12- and 13-year-old girls (r = −.31) and boys (r = −.39). Another study found that adolescent girls with low CRF exhibited 31% higher levels of depression, compared to girls with high CRF [28]. The same study also showed that boys and girls categorized as having a fit body composition exhibited 12 and 25% higher body satisfaction, respectively, compared to students categorized as having an unfit body composition. Regarding muscular strength, Lubans and Cliff [29] found an association with self-worth in boys but not girls, and a review by Smith et al. [30] showed a strong association with self-esteem in adolescent populations.

### Aim

A small amount of evidence regarding adolescents indicates a relationship between the components of health-related physical fitness and mental health outcomes. However, to the best of our knowledge, only the study by Yeatts, Martin and Petrie [31] has measured the three components CRF, muscular strength and body composition in association with a mental health outcome in adolescents. Thus, it is unclear whether one component is more important than others. Regarding Norwegian adolescents specifically, only one study has examined a mental health outcome in association with physical fitness [32]. Therefore, the aim of the present paper was to investigate the relationship between health-related components of physical fitness and mental health in Norwegian adolescents.

## Methods

### Design and participants

**Fig. 1:**
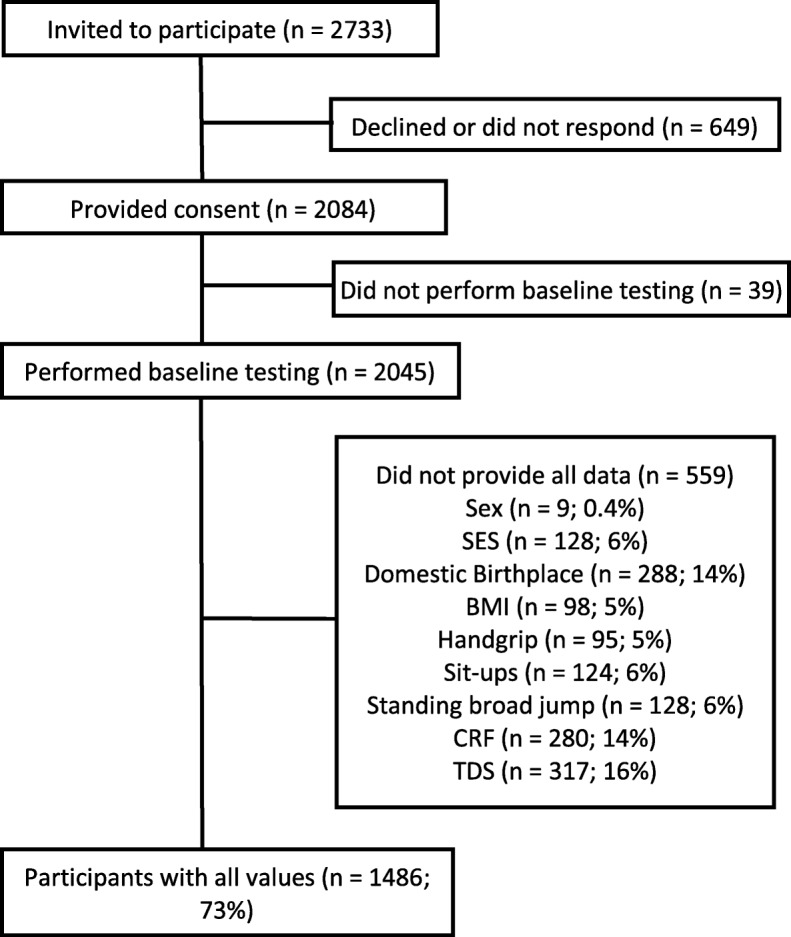
Flow chart of recruitment and participation with an overview of missing values. SES = socioeconomic status. BMI = body mass index. CRF = cardiorespiratory fitness. TDS = total difficulties score

The present study used cross-sectional data from the baseline tests of the School in Motion project [33]. This was a multicenter study, involving four geographically separate regional test centers in Norway. Out of 103 invited lower secondary schools, 29 schools agreed to participate. Only eighth grade students (13–14-year-olds) were invited to participate in the study (*n* = 2733). Informed parental consent was obtained from 76% of the invited students (*n* = 2084). Not all students had valid measures on all variables and Fig. 1 shows an overview of the participant flow. The participants were tested in the spring of 2017, during school time, at their respective schools. All test personnel received the same training beforehand to make sure there were no discrepancies in how the tests were carried out. All test procedures were approved by the Norwegian Centre for Research Data (project number 49094), and the project is in accordance with the Declaration of Helsinki for experiments involving humans.

### Measurements

#### Body composition

Participants’ weight without shoes was measured by digital scale (Seca 899, Hamburg, Germany) and all measurements were recorded to the closest 0.1 kg. Their clothes were noted, and their weight adjusted in the following analysis: 1 kg was subtracted for pants and/or sweater, 0.5 kg was subtracted for shorts/tights and t-shirt. Height was measured by portable stadiometer (Seca 123, Hamburg, Germany) and was recorded to the closest mm. The values were used to calculate individual body mass index (BMI) scores (kg/m^2^). None of the measurements were disclosed to the participants.

#### Muscular strength

Sit-ups (n/30 s), standing broad jump (best of two attempts) and handgrip test (best of two attempts), as described in the EUROFIT test battery [34] were used to measure muscular strength. Participants performed sit-ups with their knees in a 90-degree angle and their fingers locked behind their head, and their feet held to the floor by test personnel. To get a valid count, the participants had to touch their knees with their elbows, going up, and touch the floor with their shoulders, going down. Participants performed standing broad jump by jumping as far as they could from a stand still position, and the distance was recorded from the heel closest to the starting point. Measurements were recorded to the closest cm. The handgrip strength test was executed with the participants’ dominant hand, as they held their arm down alongside their body, gripping a Baseline dynamometer (Baseline® Hydraulic Hand Dynamometer, Elmsford, NY, USA) as hard as they could for 3 s. Measurements were recorded to the closest kg.

#### Cardiorespiratory fitness

CRF was assessed by a 10-min intermittent running test [35]. The test was performed by the participants running between two marked lines, 16 m apart, inside a gymnasium. They were required to touch the floor behind the line with one hand before turning and running back. The participants ran for 15 s, then paused for 15 s on the test leader’s whistle and this procedure was repeated for 10 min. According to test protocol, the intended distance between the lines is 20 m; however, limited space in many school gymnasiums compelled us to set a new standard distance at 16 m. Because of this, we could not estimate maximum oxygen uptake from the test results, therefore, we use running distance in meters (m) as an indirect measurement unit of CRF when describing our results.

#### Mental health

To measure mental health, the participants completed a Norwegian language version of the Strengths and Difficulties Questionnaire [SDQ [36];]. The questionnaire consists of 25 items divided into five subscales. The five subscales cover emotional symptoms, conduct problems, hyperactivity, peer relationships and prosocial behavior. The questionnaire contains statements such as “I worry a lot”,” I am easily distracted, I find it difficult to concentrate” and” Other people my age generally like me”. Participants reply to the statements on a three-point Likert scale:” not true”,” somewhat true” and” certainly true”. Each subscale scores from 0 to 10. Except for the prosocial subscale, a higher score signifies a higher degree of difficulties. A high score on the prosocial subscale signifies social strengths. The scores from all subscales except the prosocial are summed to create the total difficulties score (TDS). TDS scores from 0 to 40 and is a dimensional measure of mental health for children and adolescents, which means that on a population level, there is a detectable reduction in psychopathology for each point-reduction on the scale [37]. It therefore represents an indication of the general mental health state in the measured population, but in the continuation of the paper, we will refer to the outcome as either TDS or psychological difficulties. The psychometric properties of the SDQ have been validated in several countries [38–40], including Norway [41].

#### Covariates

Other variables associated with mental health are sex [42], domestic or foreign birthplace [43], and socioeconomic status [SES [44];]. The participants’ sex was noted by test-personnel, and birthplace (“Were you born in Norway”) was assessed in the questionnaire. Parents’ education level was included as a measure of SES [45].

### Statistical analysis

#### Data management

Data were managed and analyzed in IBM SPSS Statistics 25 (IBM, Armonk, New York, USA). SDQ data were scored according to the syntax provided by the SDQ information web page [46]. The syntax summed the scores from each of the four subscales needed to create the TDS variable. Cronbach’s alpha was employed to assess the internal consistency of TDS and the result was .62.

We created z scores stratified for sex and BMI quartiles for handgrip strength, standing broad jump and sit-ups. The z scores were used to create one composite mean z score for muscular strength. SES was analyzed by including only the parent with the highest education level. Next, parents’ education level was categorized as either” lower secondary school or less”,” upper secondary school”, “less than four years university education” and “four years or more university education”.

Out of 2045 participants, 27% (*n* = 559; girls = 38.2%) had at least one missing value. A new grouping variable was created to analyze differences between participants with all values (*n* = 1486) and participants with missing values (n = 559). The following primary analyses were carried out on the complete-case group only, while extensive missing value analyses were conducted to examine if they influenced the primary results.

#### Complete-case primary analyses

Descriptive statistics were calculated and are presented as means and standard deviations (SD). Seven linear mixed effect models with TDS as the dependent variable were conducted. In models one to six, we assessed the separate associations between TDS and the muscular strength variables and the health-related fitness components. In the seventh model, the fitness components controlled for each other. All models controlled for the covariates (sex, domestic birthplace and SES). We report estimates (unstandardized coefficients) and their 95% confidence intervals (95% CI). Estimates reflect the change in TDS as a result of one unit of measurement increase in the independent variables. Initial linear mixed effect modelling showed no statistically significant interaction effects between sex and the physical fitness variables, using TDS as the dependent variable. To account for possible effects of clustering of observations within schools, school site was included as a random effect in all models. A *p* value < .05 indicated statistical significance.

#### Missing value analyses

To assess whether missing values were missing completely at random (MCAR), Little’s MCAR test was used. The analysis did not support MCAR (104.331, DF = 24, *p* < .001). Pattern analysis (not shown) indicated that the data were likely missing at random (MAR). A possible explanation for the missing values is that we never forced the participants to complete the tests, which may have caused some participants to opt out. For instance, many stated that they did not want to run the CRF test. Moreover, the SDQ was one of many components in a large and extensive questionnaire. The missing data from the SDQ may be a consequence of the size and duration of the extended questionnaire, which may have caused many to quit before completion. However, this is unclear and there may be other reasons unknown to us.

One-way ANOVA was used to assess differences between the complete-case group and the missing-values group. Pearson’s correlation analysis was used on the fitness variables and TDS, for the purpose of examining if associations were similar in both groups. Multicenter studies are vulnerable to differences in missingness between test centers [47], and this was examined using frequency statistics. As our final action in handling the missing values, we employed multiple imputation [48, 49]. Five imputations were generated from relevant variables, using the automatic procedure with 10 iterations, with the assumption that data were missing at random. A linear mixed effects model was conducted on the imputed dataset, with TDS as the dependent variable, and all health-related components of physical fitness variables and covariates entered as independent variables. The imputed dataset results are presented, in addition to the complete-case results, as recommended by Manly and Wells [50] and Sterne et al. [51].

## Results

### Descriptives and group comparisons

Descriptive results and group differences between the complete-case group and missing-values group are presented in Table 1. Compared to the complete-case group, the missing-values group had 1.5% higher mean BMI (*p* = .047), performed 5.9% worse on the handgrip test (*p* < .001), 2.3% worse on the standing broad jump test (*p* = .005) and had 2.9% higher CRF (p < .001). The missing-values group scored 15.5% higher for TDS (p < .001).

**Table 1:** Participant mean characteristics from the complete-case group and the missing-values group. Presented as means with standard deviations (SD)

	Total difficulties score (0–40)	Body mass index	Sit-ups (n/30 s)	Standing broad jump (cm)	Handgrip strength (kg)	Cardiorespiratory fitness (meters)
**All values present**						
Yes (n = 1486)	10.3 (5.1)	19.8 (3.1)	19 (4)	172.2 (25.9)	30.5 (7.0)	906 (98)
No (*n* = 242–559)	11.9 (5.7)^a^	20.2 (3.5)^a^	19 (4)	168.2 (27.8)^a^	28.7 (7.4)^a^	932 (115)^a^

*Note.*^a^Difference between groups is significant at *p* level < .05

### Linear mixed effects models

Results from the linear mixed effects models are summarized in Table 2. Model 1 indicated no association between BMI and TDS. Model 2 indicated an inverse association between sit-ups and TDS (b = −.088; 95% CI = −.156 to −.020; *p* = .011). Model 3 indicated no association between standing broad jump and TDS. Model 4 indicated no association between handgrip strength and TDS. Model 5 indicated an inverse association between muscular strength and TDS (b = −.458; 95% CI = −.810 to −.109; *p* = .010) Model 6 indicated an inverse association between CRF and TDS (b = −.006; 95% CI = −.009 to −.003; *p* < .001). The fully adjusted model (7) with all independent variables and covariates entered simultaneously, revealed no association between muscular strength and TDS, while the association between CRF and TDS remained almost identical as in Model 6 (b = −.006; 95% CI = −.009 to −.002; *p* = .001). The estimates produced by the fully adjusted model suggest that every 100 m distance increase in the CRF test is associated with 1.5% (0.6 points) lower TDS.

**Table 2:** Linear mixed effect models with Total difficulties score as the dependent variable

Model	Independent variable	B (95% CI)	p
**1**	Body mass index	.058 (−.027 to .142)	.184
**2**	Sit-ups	−.088 (−.156 to −.020)	.011
**3**	Standing broad jump	−.008 (−.018 to .003)	.147
**4**	Handgrip strength	−.024 (−.062 to .013)	.206
**5**	Muscular strength z score	−.458 (−.810 to −.109)	.010
**6**	Cardiorespiratory fitness	−.006 (−.009 to −.003)	<.001
**7 – Fully adjusted model**	Intercept	18.8 (14.6 to 23.0)	<.001
	Body mass index	.003 (−.086 to .093)	.941
	Muscular strength z score	−.033 (−.450 to .381)	.873
	Cardiorespiratory fitness	−.006 (−.009 to −.002)	.001

*Note.* 29 school clusters were included as random effects in all models; all models were controlled for sex, socioeconomic status, and birthplace. *B* Unstandardized regressioncoefficient, *CI* Confidence interval, *p* Significance level

### Results from missing value analyses

Among the 559 excluded participants, 12.2% (*n* = 68) had completed the SDQ and performed the CRF test. Correlation analysis indicated a non-significant, inverse correlation between CRF and TDS (r = −.13; *p* = .139), a similar relationship as in the complete-case group. Other correlations were also similar (data not shown), indicating a small likelihood of systematic differences between the complete-case group and missing-values group.

There was a difference in missingness between test centers. One test center represented 10.7% of the participants in the complete-case group and 30.4% in the missing-values group, which means that more than half of all participants from this region were excluded due to missing values. This was caused by low completion of the SDQ, with only 13.5% of these participants completing the SDQ in the missing-values group. The corresponding completion rates from other test centers were 51.2–67.6%.

Finally, the pooled dataset from the multiple imputation (*n* = 2045) was analyzed with a fully adjusted linear mixed effects model, in the same way as the complete-case dataset. The association between CRF and TDS was close to identical to the complete-case results (b = −.006; 95% CI: −.010 to −.002; *p* = .006). Thus, all missing-value analyses indicated that the results likely would have been unchanged with all values present.

## Discussion

The main findings of the present study were that higher CRF was significantly associated with lower psychological difficulties in Norwegian adolescents while controlling for muscular strength, body composition, socioeconomic status, school clustering, sex and domestic/foreign birthplace. The results indicated that psychological difficulties were not associated with muscular strength or body composition, when controlling for the aforementioned variables.

### Muscular strength, body mass index and metal health

A significant association between muscular strength and TDS was initially found. However, when controlling for CRF there was no association between these variables. A possible explanation is that the participants with high CRF were also likely to have a relatively high muscular strength [52]. However, based on the fully adjusted model, it can be postulated that participants with high muscular strength did not necessarily have high CRF. A possible interpretation is that muscular strength in adolescents is generally a natural consequence of the individual’s CRF level, which may represent the true association with psychological difficulties.

The present findings support previous studies that have suggested CRF to be the only health-related aspect of fitness associated with mental health outcomes such as quality of life [53], depression [31, 54] and well-being [55]. Many studies that have found associations between muscular strength and mental health outcomes in adolescents did not measure CRF [29, 56]. The present findings did not show an association between BMI and psychological difficulties, independent from controlling for CRF. This is congruent with the review by Luppino et al. [21], who found an association between overweight and depression in adults, but not in individuals younger than 20 years. This indicates a different relationship between age groups; however, it is important to point out that none of the reviewed studies controlled for CRF. Although it is possible that muscular strength and BMI are associated with mental health outcomes, studies that do not also measure CRF lack important information. Had we not controlled for all fitness variables in the present study, we would have erroneously concluded that muscular strength was associated with TDS. Opposing findings by Kettunen et al. [57] showed muscular strength to be more important than CRF to reduce stress in adults. However, this study categorized continuous variables and employed ANOVAs, which has been strongly advised against by Altman and Royston [58] and might have produced biased results. Additionally, associations between physical fitness and mental health might be different in adult and adolescent populations. For instance, many experimental studies have found effects of strength training on mental health in older adults [23]. Positive effects in older adults are not surprising, considering how strength training can reverse muscle atrophy and improve the daily functioning of older people [59]. In adolescents however, muscular strength is mainly associated with appearance-related mental health outcomes, such as self-perception, perceived physical appearance or physical self-worth [30]. Future studies of associations between health-related physical fitness and mental health should include different mental health outcomes, to gain a better understanding of whether specific components of fitness are associated with specific outcomes of mental health.

### Cardiorespiratory fitness and mental health

Although a causal direction between CRF and psychological difficulties cannot be established from cross-sectional findings, recent evidence has indicated a one-directional causal relationship for physical activity as a protective factor against depression among adults [60]. High-intensity exercise is an important factor for high CRF [18], hence results from the present study support a hypothesis suggesting that high-intensity exercise might be more favorable for mental health than low-intensity exercise. This is in accordance with the study by Parfitt, Pavey and Rowlands [61], who found high-intensity exercise to be more favorable for mental health than light-intensity exercise, in a population of children. Furthermore, the meta-analysis by Ahn and Fedewa [62] found high-intensity exercise RCT interventions to have the most effect on children’s mental health. On the other hand, Helgadóttir et al. [63] concluded that low-intensity exercise was more effective on depression treatment than high-intensity exercise in an adult population. The low-intensity group exercised with yoga and this type of exercise may have a distinct relationship with mental health. However, the results should be treated with caution, because the intervention had low adherence and did not mention how this differed between exercise groups. Additionally, 12 months after the intervention, there were no significant differences between the low- and vigorous-exercise groups. The study by Helgadóttir et al. [63] is incongruent with the previously mentioned studies, as well as what Bailey et al. [64] suggested to treat depression in adolescents: “… aerobic-based activity of moderate-to-vigorous intensity.” It is also possible that intensity might not even be especially crucial, as long as CRF is improved. Shepherd et al. [65] prescribed high-intensity interval training and moderate-intensity continuous training in two groups of inactive adults and both groups experienced increased CRF and improved mental health. Few studies have examined the causal relationship between increased CRF and improved mental health outcomes, but a recent longitudinal study by Rahman et al. [66] showed that improved CRF predicted at least a 50% reduction in depression scores for adults. Ruggero et al. [27] found that high CRF at baseline was associated with lower levels of depression a year later in adolescent girls and suggested that CRF might mediate the effect physical activity has on depression. This was supported by Eddolls et al. [67] who concluded that CRF mediated the relationship between vigorous physical activity and mental health in adolescents, thus suggesting that physical activity interventions to treat depression may only be effective if they improve CRF.

Research on the potential explanatory mechanisms between muscular strength and mental health is scarce [23]. There are, however, mechanisms that might explain the association between CRF and psychological difficulties. One example is the endocannabinoid system, which mediates high-intensity aerobic exercise effect on depression [68]. Psychosocial mechanisms may also have had a mediating role in the present results: CRF is associated with team sports like football, handball and basketball [69], which are important arenas for social relationships and can provide opportunities to improve self-esteem and body satisfaction [12]. The topic of explanatory mechanisms between physical fitness and mental health outcomes requires more research, especially on adolescent populations, in order to fully understand the relationship between the relevant variables. Additionally, future studies need to examine how exercise at different intensities affects different mental health outcomes, and whether the results are influenced by increases in CRF. Such knowledge can be useful in efforts to prevent or treat mental disorders.

### Strengths and limitations

Strengths of the present study include the large sample size from separate geographical regions, the use of three objectively measured health-related components of physical fitness, and the control of relevant covariates.

The main limitation of the present study was a large number of missing values; however, the extensive missing value analyses indicated that the main results most likely were unaffected by the dropouts. Reducing the length of the CRF-test distance is also a limiting factor, as it makes our CRF-results incomparable to results from other studies. However, this limitation did not affect the main results since running distance in meters was used as the measurement unit, and not estimated maximum oxygen uptake. Moreover, we did not measure maturity status, which may act as a confounder for the associations in the main results.

The present internal consistency results pertaining to SDQ were quite low and are similar to Italian [70], Finnish [71] and Dutch [40] results. Internal consistency results from English speaking populations [72, 73] are usually higher, which suggests that statements are better understood by native English speakers, while non-native English speakers may misinterpret the statements somewhat. Age is also a factor, as the internal consistency is lower for younger adolescents, such as the present population, compared to older adolescents as examined in studies by Bøe et al. [74] and Sagatun et al. [75]. Finally, the cross-sectional nature of the study limits the ability to make any causal inference.

## Conclusion

The main findings from the present study was that higher cardiorespiratory fitness was significantly associated with lower levels of psychological difficulties in adolescents. Body composition was not associated with psychological difficulties. Muscular strength was separately associated with psychological difficulties but not when controlling for cardiorespiratory fitness. This indicates that strength training or focus on weight reduction may be ineffective in efforts to prevent or treat mental health problems in adolescents. Future research in this area should examine the prospective associations between physical fitness components and mental health outcomes and explore potential reasons why cardiorespiratory fitness seems to be more important for adolescent mental health outcomes than muscular strength and body composition.

## Data Availability

The datasets generated and/or analyzed during the current study are not publicly available as publications are planned but are available from the corresponding author on reasonable request.

## Abbreviations

CRF: Cardiorespiratory fitness
BMI: Body mass index
SDQ: Strengths and Difficulties Questionnaire
TDS: Total Difficulties Score
SES: Socioeconomic status
MCAR: Missing completely at random
MAR: Missing at random
